# Not all babies are the same: an examination of temperament profiles during infancy

**DOI:** 10.3389/fpsyg.2026.1727489

**Published:** 2026-03-17

**Authors:** Catherine Meloche, Magdalena A. Zdebik, Jean-Pascal Lemelin, Jessica Pearson

**Affiliations:** 1 University of Quebec in Outaouais, Gatineau, QC, Canada; 2 Centre de Recherche Universitaire sur les Jeunes et les Familles (CRUJeF), Québec, QC, Canada; 3 Groupe de Recherche sur l'Inadaptation Psychosociale chez l'Enfant (GRIP), Montréal, QC, Canada; 4 Sherbrooke University, Sherbrooke, QC, Canada; 5 University of Quebec at Trois-Rivières, Quebec, QC, Canada

**Keywords:** child development, infancy, latent profile analysis, socioemotional development, temperament

## Abstract

Early temperament plays an essential role in shaping children’s reactions to their environment and can therefore have important implications for socioemotional and cognitive development throughout childhood. Investigating how different temperament characteristics group together in infancy can identify how distinct temperamental profiles are represented in the general population and potentially offer valuable insights into long-term developmental outcomes. However, few studies have identified temperament profiles in infants. The objective of this study was to identify temperament profiles using the 14 dimensions described in the Rothbart’s model of infant temperament in a general population sample of 6-month-old infants. Participants were 433 French-speaking mothers of low-risk socioeconomic background who assessed their child’s temperament at 6 months of age using the *Infant Behavior Questionnaire-Revised short form*. Latent profile analysis revealed four distinct temperament profiles among the infants: (1) *Moderate Reactivity and Regulation* (37.67%); (2) *High Positivity-High Regulation* (28.64%); (3) *High Negativity-Low Regulation* (13.60%); and (4) *High Positivity and Negativity* (20.09%). These profiles offer a more nuanced understanding of early temperament and highlight that complex individual configurations are present at a very young age. Prevention and intervention programs targeting infants and their parents should take these temperament profiles into consideration.

## Introduction

Infant temperament plays a crucial role in shaping children’s reactions to their environment and can have significant implications for their overall development and psychosocial adjustment (Morales et al., 2021; Paulus et al., 2022; Wu et al., 2022). Examining temperament profiles in early life can help identify children who may be at risk for later socioemotional and behavioral problems (Rothbart and Putnam, 2002). Despite this, relatively few studies have identified temperament profiles in infancy (Augustine et al., 2022; Beekman et al., 2015; Costa and Figueiredo, 2011; Gartstein et al., 2017; Planalp and Goldsmith, 2020).

Children can respond to the same environmental challenges in very different ways, depending on their temperament (Chess and Thomas, 1996). Investigating how different characteristics cluster together in very young children can help identify distinct temperamental profiles and determine how these profiles are represented in the general population. A better understanding of infant temperament could therefore potentially offer valuable insights to help tailor prevention and intervention programs to promote child development.

### Temperament

Temperament is characterized by biologically based individual differences in reactivity and self-regulation observable early in life and influenced by heredity, maturation, and experience (Goldsmith et al., 1987; Rothbart, 2007; Rothbart and Derryberry, 1981; Shiner et al., 2012). Reactivity describes an individual’s responsiveness to changes in both external and internal environments, including a wide array of emotional and physiological reactions measured by the latency, duration, and intensity of responses such as fear, anger, and positive affect (Rothbart and Bates, 2006). Self-regulation involves processes that modulate reactivity, enabling individuals to manage the intensity and duration of their emotional and behavioral responses (Rothbart and Bates, 2006).

Rothbart’s theoretical framework is currently the most widely used in child temperament research due to its comprehensive approach, strong empirical support and the validity of its measurement instruments (Shiner et al., 2012; Rothbart, 2012; Rothbart and Derryberry, 1981). Rothbart’s model (1987) emphasizes not only behavioral and emotional reactivity but also the attentional and behavioral processes that regulate these initial reactive predispositions. During infancy, the model describes 14 distinct dimensions of temperament that can be grouped into three broad factors: the Surgency/Extraversion and the Negative Affectivity factors both refer to emotional and behavioral reactivity, while the Orienting/Regulation factor involves regulatory processes (Gartstein and Rothbart, 2003; Rothbart and Bates, 2006). Surgency/Extraversion reflects children’s levels of sociability, positive affect, activity and sensation seeking. It includes temperament dimensions of Approach, Vocal Reactivity, High Intensity Pleasure, Smiling and Laughter, Activity Level, and Perceptual Sensitivity. The Negative Affectivity factor refers to children’s tendency to react toward new, unpleasant, or potentially threatening situations with various negative emotions (e.g., fear, anger, sadness, distress) and the tendency to take longer to recover from heightened emotional reactivity. It encompasses dimensions such as Sadness, Distress to Limitations, Fear, and Falling Reactivity. Finally, the Orienting/Regulation factor corresponds to children’s ability to focus attention and to inhibit behaviors in order to adapt to environmental demands, and at later ages is known as Effortful Control. It is characterized by dimensions such as Low Intensity Pleasure, Cuddliness, Duration of Orienting, and Soothability. While temperament dimensions can be grouped into these three major factors, studying each dimension individually allows for a more fine-grained understanding and greater variability in describing infants’ temperament.

Table 1 describes Rothbart’s 14 dimensions of infant temperament, as presented in the *Infant Behavior Questionnaire* (IBQ; Rothbart, 1981) and its revised version (IBQ-R; Gartstein and Rothbart, 2003) that are designed to measure temperament in infants between the ages of 3 and 12 months.

**Table 1:** Dimensions of the infant behavior questionnaire (IBQ-R; Gartstein and Rothbart, 2003).

Dimension	Description
Approach	Rapid approach, excitement, and positive anticipation of pleasurable activities.
Vocal reactivity	Amount of vocalization exhibited by the baby in daily activities.
High intensity pleasure	Pleasure or enjoyment related to high stimulus intensity, rate, complexity, novelty, and incongruity.
Smiling and laughter	Smiling or laughter during general caretaking and play.
Activity level	Gross motor activity, including movement of arms and legs, squirming and locomotor activity.
Perceptual Sensitivity	Detection of slight, low intensity stimuli from the external environment.
Sadness	Lowered mood and activity related to personal suffering, physical state, object loss, or inability to perform a desired action; general low mood.
Distress to limitations	Fussing, crying or showing distress while (a) in a confining place or position; (b) in caretaking activities; (c) unable to perform a desired action.
Fear	Startle or distress to sudden changes in stimulation, novel physical objects or social stimuli; inhibited approach to novelty.
Falling reactivity	Rate of recovery from peak distress, excitement, or general arousal; ease of falling asleep.
Low intensity pleasure	Amount of pleasure or enjoyment related to low stimulus intensity, rate, complexity, novelty and incongruity.
Cuddliness	Expression of enjoyment and molding of the body to being held by a caregiver.
Duration of orienting	Attention to and/or interaction with a single object for extended periods of time.
Soothability	Reduction of fussing, crying, or distress when soothing techniques are used by the caregiver.

### Importance of early temperament research

A relatively substantial number of studies has examined how specific temperament traits may predispose children to specific adjustment issues. For instance, shyness and fear have been associated with internalizing problems, while high intensity pleasure and low effortful control were linked to externalizing problems (Oldehinkel et al., 2004). Moreover, negative reactivity was found to be associated with externalizing behavior problems, while distress to novelty was linked to internalizing problems such as anxiety (Krieger and Stringaris, 2016; Sandstrom et al., 2020). Furthermore, strong regulatory abilities have been identified as a protective factor for both internalizing and externalizing problems (Nielsen et al., 2019). Regarding school readiness and academic success, Gobeil-Bourdeau et al. (2022) found that child temperament significantly interacted with various aspects of the family environment to predict school readiness. For example, children with high effortful control benefitted more from positive parenting, exhibiting higher receptive vocabulary skills. On the other hand, low effortful control was negatively affected by sociodemographic risk, with links to lower levels of basic knowledge and social adjustment. Associations between temperament and cognitive development have also been reported. Lower activity levels in infancy were associated with higher cognitive outcomes at 36 months, while traits like anger proneness and interest persistence showed context-dependent associations influenced by psychosocial risk levels (Lemelin et al., 2006). More precisely, anger proneness was negatively associated with cognitive development in the low-psychosocial-risk group, whereas this association was not significant in the high-risk group. Regarding interest persistence, a positive association with cognitive development was found in the low-risk group, while this association was negative in the high-risk group. Moreover, Karrass and Braungart-Rieker (2004) highlighted that distress to novelty was a significant predictor of cognitive outcomes, where higher levels of distress to novelty at 4 months were linked to higher IQ scores at 36 months.

However, examining distinct temperament traits individually could lead to conflicting results regarding their associations with child outcomes. Ermanni and Bell (2025) highlight that surgency has been associated with both externalizing problems and social competence. Such discrepancies may be attributable to cases in which individuals exhibit similar levels of surgency but differ substantially on other temperamental dimensions, suggesting that associations with outcomes could be better understood by considering multiple temperament dimensions simultaneously. Other studies, despite their small number, have shown that certain configurations of temperament traits (i.e., temperament profiles) have also been differentially linked to internalizing or externalizing problems (van den Akker et al., 2010). Notably, toddlers classified within a “fearful” profile, characterized by high levels of social fear and intermediate levels of anger proneness and activity, tended to experience higher levels of internalizing problems such as anxiety and withdrawal. On the other hand, children with an “expressive” profile, marked by low social fear and high levels of both anger proneness and activity, were more susceptible to externalizing problems, including aggression and hyperactivity. Furthermore, temperament profiles varying in self-regulation abilities were linked to academic success (Ato et al., 2020). In Ato et al. (2020) study, school-aged children who were classified in a “Sociable/High Regulation” temperamental profile exhibited higher levels of self-regulation and tended to achieve greater academic performance and social acceptance. In contrast, children classified in the “Emotionally Negative/Low Regulation” profile were more likely to experience academic and social difficulties, such as a higher likelihood of peer rejection and lower performance in language and mathematics. The few studies that have examined children’s adjustment in relation to temperament profiles suggest that gaining a deeper understanding of the configurations of temperament traits early in life would offer valuable insights into the long-term development of temperament and its associated outcomes.

### Temperament profiles

#### Pioneering research in temperament profiles

Contemporary theory and research on infant and child temperament and its impact on emotional functioning and behavioral adjustment are grounded in the pioneering work of Chess and Thomas (1996; Thomas, 1977). They were the first to propose that children’s temperament can be classified into different categories, allowing for a better understanding of the common configurations of various temperamental traits. They identified nine temperament traits (activity level, rhythmicity, approach/withdrawal, adaptability, intensity, mood, persistence/attention span, distractibility, and sensory threshold) that lead to unique combinations to form a child’s individual temperament, which were categorized into three primary styles: easy, difficult, and slow-to-warm-up (Thomas, 1977). They observed that approximately 40% of infants displayed an easy temperament, characterized by regular routines, a cheerful disposition, adaptability, and an easygoing nature. Another 10% of infants were deemed to have a difficult temperament, frequently exhibiting irregular routines, problematic sleep cycles, slow adaptation to new circumstances, and intense negative reactions to changes and new people. Approximately 15% of the infants were categorized as slow-to-warm-up, typically showing low activity levels, reluctance in new situations, and slow adjustment to changes, with a generally negative mood. The remaining 35% of infants did not fit entirely into any one category, often displaying a mix of these temperament styles.

Building on the pioneering work of Chess and Thomas, Rothbart (1981) developed a parent-report instrument with high internal reliability that captures aspects of reactivity and self-regulation, which are considered conceptually distinct from each other (Rothbart and Bates, 1998). Rothbart’s perspective emphasizes the variety of temperament dimensions, providing a more detailed understanding of individuals as complex systems that cannot be fully captured by isolating individual variables. These varied dimensions allow for the identification of common temperament-based subgroups, offering pathways to more targeted risk assessment and intervention (Hawes et al., 2021).

#### Latent profile analysis in temperament studies

As researchers gain a deeper comprehension of the broad spectrum of temperament dimensions, temperament profiles are increasingly being used to understand human behavior (Goldsmith et al., 1987). The current study employed latent profile analysis (LPA; Muthén and Muthén, 2000), a statistical method that uses a person-centered approach to identify distinct patterns in the co-occurrence of continuous dimensions of temperament across individuals. LPA offers significant advantages over traditional clustering techniques by using continuous indicators in a statistical model, which avoids arbitrary cutoffs and allows to handle missing data (Bergman and Magnusson, 1997; Muthén, 2004). Additionally, LPA provides probability scores for each profile, accounting for membership uncertainty and enabling more precise identification of temperament patterns (Gartstein et al., 2017; van den Akker et al., 2010). Individuals are allocated to the profile they are most likely to belong to, allowing for a more objective and precise understanding of how dimensions of temperament co-occur across individuals (Beekman et al., 2015).

Previous studies using LPA in temperament studies have typically identified between three and five latent profiles (Augustine et al., 2022; Beekman et al., 2015; Dalimonte-Merckling and Brophy-Herb, 2019; Dollar et al., 2017; Ermanni and Bell, 2025; Gartstein et al., 2017; Laible et al., 2014; Planalp and Goldsmith, 2020; van den Akker et al., 2010). For instance, van den Akker et al. (2010) analyzed maternal ratings of child sociability, activity level, and anger proneness using the *Toddler Behavior Assessment Questionnaire* (TBAQ; Goldsmith, 1996) and identified three profiles: “Typical,” “Expressive,” and “Fearful.” The “Typical” profile was characterized by lower levels of social fear, anger proneness, and activity level and was associated with well-adjusted behaviors. The “Expressive” profile exhibited the highest levels of anger proneness and activity level, along with intermediate levels of social fear. Children in this profile were both anger-prone and active. The “Fearful” profile was marked by the highest levels of social fear and intermediate levels of anger proneness and activity level. The stability of these profiles was high, with approximately 71% of children retaining their profile membership from the first (T1, mean age = 30 months, *SD* = 6.5, range = 18–43 months) to the second time point (T2, mean age = 36 months, *SD* = 6.5, range = 26–49 months) of the study.

Furthermore, Beekman et al. (2015) also employed LPA to analyze child temperament across multiple assessment points. When infants were 9 months old, five scales of the IBQ (Rothbart, 1981) were used: activity level, distress to limitations, distress to novelty, duration of orienting and smiling and laugher. Corresponding dimensions of temperament were assessed at age 18 and 27 months using the TBAQ (Goldsmith, 1996), including activity level, anger proneness, fear, interest and pleasure. Four profiles were identified from infancy to toddlerhood: “Typical Low Expressive,” “Typical Expressive,” “Negative Reactive,” and “Positive Reactive.” At 9 months, the “Typical Low Expressive” profile was characterized by slightly below-average values across all temperament dimensions, while the “Typical Expressive” profile showed slightly above-average values. The “Negative Reactive” profile displayed well above-average activity level, distress to novelty and limitations, and below-average levels of smiling, laughter, and soothability. The “Positive Reactive” profile had below-average activity level and distress to limitations, with above-average levels of smiling, laughter, and soothability. These profiles remained consistent at ages 18 and 27 months.

Moreover, a study using data from the NICHD Study of Early Child Care and Youth Development (Laible et al., 2014) identified temperament profiles in children at 54 months. Based on maternal ratings of negative emotionality (sadness, anger and fear) and effortful control (a composite of inhibitory control and attention) assessed with the *Child Behavior Questionnaire* (Rothbart et al., 2001), four profiles emerged: “Moderate Regulation and Moderate Negative Emotionality,” “Very Low Regulation and High Anger Emotionality,” “Low Regulation and High Negative Emotionality,” and “High Regulation and Low Negative Emotionality”. These profiles demonstrated significant stability over time, with children maintaining their profile characteristics through first and third grade. The study also revealed that children with a “High Regulation and Low Negative Emotionality” profile were rated as the most prosocial and cooperative by parents and teachers.

### Study rationale and objective

Although temperament profiles identified using LPA have been explored in previous literature, the current study aims to build on this body of work and address some limitations. First and foremost, relatively few studies focus on the general population compared to more numerous studies that have examined clinical samples but that may not generalize to a broader context. Previous research has examined temperamental profiles based on a limited number of temperament dimensions. In the current study, we consider 14 dimensions of temperament simultaneously, in order to capture the complexity and descriptive variability of child temperament based on Rothbart’s model of temperament. Additionally, most studies assessed older children as opposed to infants. The present study examines temperament profiles early in a child’s life: at 6 months of age. Examining temperament at such a young age is not only relatively rare but also important considering the stability of temperament traits over time. Moreover, research on very young children in a French-Canadian population is notably lacking, limiting our ability to explore potential cultural differences in temperament (Putnam et al., 2024). Identifying temperament profiles early in life could provide significant insights into how families adapt to life with a new child who has unique characteristics and could help to normalize the diverse manifestations of temperament that can be observed in infants, hence potentially reducing parental stress.

The objective of the present study was to identify temperament profiles using the 14 dimensions described in the Rothbart’s model of infant temperament. In the current study, LPA was used to examine early temperament profiles in a general population sample of 6-month-old infants.

## Materials and methods

### Participants and procedure

The sample consists of 433 French-speaking mother–child dyads from the province of Quebec, Canada, who are part of a longitudinal study on child development. Recruitment was conducted through social media (e.g., pregnancy-related Facebook pages or prenatal resources) and by emails sent through regional daycare centers. Inclusion criteria were that participants lived with their child (with regular contact exceeding 40% of the time) and had a child without any condition or illness significantly affecting their development. The project was approved by the Human Research Ethics Committee of the University of Quebec at Trois-Rivières (CER-20-271-07.03).

Socio-demographic data were collected from the participants during pregnancy (T0). When their child was 6 months old, mothers completed an online questionnaire (T1) evaluating the child’s temperament. Participants who completed the temperament questionnaire at T1 were included in this study. No significant differences were found on various sociodemographic variables between participants who did or did not complete the temperament questionnaire (maternal age, maternal highest level of education, family income, marital status, ethnicity and child sex and age; all *p*s > 0.05).

### Measures

#### Infant temperament

Infant temperament was measured using the *Infant Behavior Questionnaire-Revised-Short Form* (IBQ-R-Short Form, Putnam et al., 2014), an abbreviated version of the updated version (IBQ-R, Gartstein and Rothbart, 2003) of the original IBQ (Rothbart, 1981), created to assess temperament in infants aged 3 to 12 months. The IBQ-R-Short Form contains 91 items assessed on a 7-point Likert scale ranging from 1 “never” to 7 “always” and includes a “not applicable” option. The 14 dimensions of temperament (see Table 1) that are measured can be grouped into three broad factors: Surgency/Extraversion, Negative Affectivity, and Orienting/Regulation. The Surgency/Extraversion factor includes six dimensions: Approach, Vocal reactivity, High Intensity Pleasure, Smiling and Laughter, Activity Level, and Perceptual Sensitivity. The Negative Affectivity factor includes four dimensions: Sadness, Distress to Limitations, Fear, and Falling Reactivity (reverse-coded). Lastly, the Orienting/Regulatory factor includes four dimensions: Low Intensity Pleasure, Cuddliness, Duration of Orienting, and Soothability. For each dimension, a mean score is calculated, where higher scores reflect greater levels of the dimension in question.

The short form of the IBQ-R demonstrates strong psychometric properties (Putnam et al., 2014). Convergent validity was assessed between the original and short versions of the IBQ-R, showing strong correlations for the dimensions of the short version with those of the original (α = 0.63 to 0.86; Putnam et al., 2014). Internal consistency for the dimensions in this sample varied between α = 0.67 and 0.80.

### Statistical analyses

Descriptive statistical analyses were performed using SPSS 28 software (IBM Corp, 2021) and main analyses were performed using MPlus Version 8.10 (Muthén and Muthén, 2000). LPA was conducted using the 14 temperament dimensions of the IBQ-R (Gartstein and Rothbart, 2003). The data were transformed into *Z*-scores and the reported scores represent deviations (in standard deviations) from the sample mean. To determine the most accurate number of temperament profiles represented in our sample, we compared the Bayesian Information Criterion (BIC), the Sample Size Adjusted Bayesian Information Criterion (SA BIC), the Akaike Information Criterion (AIC), Entropy scores, the Vuong-Lo–Mendell–Rubin Likelihood Ratio Test (VLMR-LRT) and the Lo–Mendell–Rubin Adjusted Likelihood Ratio Test (LMR-LRT). Although previous studies tend to obtain three to five temperamental profiles, we tested models ranging from one to seven profiles. Models were compared to assess fit and to determine the appropriate number of groups. The indices (AIC, BIC, SA BIC) are compared based on the number of groups. The chosen indicators are those that show the smallest decrease with no further gains observed when additional groups are introduced. The final model was selected based on several criteria: the lowest AIC, BIC and SA BIC, the highest average posterior probabilities of group membership (indicating the accuracy of individuals’ assignment to each group; Nagin, 1999; Nagin, 2005), with a minimum of 80% correct classification for each profile, and adequate power for analyses (i.e., each trajectory group consisting of more than 10% of the sample). Variables were assumed to follow a normal distribution.

## Results

### Descriptive statistics

Table 2 presents the descriptive statistics for the sociodemographic variables, while Table 3 presents the means and standard deviations for the 14 dimensions of temperament measured by the IBQ-R. At T0, the average age of mothers was 29.51 years (*SD* = 3.70), and 46.4% of them were first-time mothers. The majority had a college degree (66.1%), were in a relationship with the child’s father (96.5%) and were White (87.9%). The median annual family income ranged between $100,000 and $150,000 CAD. At T1, 57.3% of the children were boys and 42.7% were girls, and the infants were on average 6.11 months old (*SD* = 0.62).

**Table 2:** Sociodemographic variables.

Sociodemographic variable	Mean (standard deviation)	*n* (%)
Maternal age	29.51 (3.70)	
Maternal highest level of education		
Primary school		3 (0.7)
Secondary school		11 (2.6)
Professional training		37 (8.6)
Collegial degree		95 (22.0)
Undergraduate degree		177 (41.1)
Master’s degree		94 (21.8)
Doctorate		14 (3.2)
Family income		
Less than $20,000		3 (0.7)
Between $20,000 and $40,000		17 (4.0)
Between $40,000 and $60,000		25 (5.9)
Between $60,000 and $80,000		50 (11.7)
Between $80,000 and $100,000		102 (23.9)
Between $100,000 and $150,000		174 (40.7)
More than $150,000		56 (13.1)
Marital status		
Single		10 (2.4)
In a relationship with the child’s father		418 (96.5)
In a relationship with another spouse		3 (0.6)
Separated or divorced		1 (0.2)
Ethnicity		
White		381 (88.0)
African Canadian		6 (1.4)
Hispanic		5 (1.1)
Asiatic		7 (1.6)
Aboriginal		1 (0.2)
Mix		9 (2.1)
Other		3 (0.7)
Prefer not to answer/Do not know		21 (4.9)
Siblings		
Yes		232 (53.6)
No		201 (46.4)
Child sex		
Boy		247 (57.3)
Girl		184 (42.7)
Child age (months)	6.11 (0.61)	

**Table 3:** Infant temperament scores on the 14 dimensions of the IBQ-R.

Temperament dimension	Mean (standard deviation)
Surgency/Extraversion	
Approach	5.23 (0.95)
Vocal reactivity	4.63 (1.01)
High intensity pleasure	5.86 (0.82)
Smiling and laughter	4.59 (1.04)
Activity level	3.89 (0.95)
Perceptual sensitivity	3.48 (1.60)
Negative affectivity	
Sadness	3.14 (1.05)
Distress to limitations	3.59 (1.10)
Fear	2.61 (1.16)
Falling reactivity (reverse coded)	5.38 (0.94)
Orienting/Regulatory capacity	
Low intensity pleasure	5.45 (0.88)
Cuddliness	6.04 (0.73)
Duration of orienting	4.30 (1.19)
Soothability	5.76 (0.82)

### Latent profile analysis

A series of latent profile models was used to examine profiles of infant temperament across 14 dimensions. Models ranging from one to seven profiles were evaluated. Comparing the different indices presented in Table 4, the four-profile model was retained. More precisely, the four-profile model had lower AIC (16006.90), BIC (16304.07), and SA BIC (16072.40) values compared to other models, a high entropy score (above 0.80), and no group consisted of less than 10% of the sample. Although more complex models had slightly lower AIC and BIC values, they did not meet the power criteria as some of their groups represented less than 10% of the sample.

**Table 4:** Latent profile analysis: model fit assessment for 1 to 7 profiles.

Indices	1-profile model	2-profile model	3-profile model	4-profile model	5-profile model	6-profile model	7-profile model
AIC	17128.67	16485.61	16176.46	16006.90	15903.15	15832.37	15778.85
BIC	17242.65	16660.65	16412.56	16304.07	16261.37	16251.66	16259.19
SA BIC	17153.79	16524.19	16228.50	16072.40	15982.11	15924.79	15884.73
Entropy	–	0.84	0.80	0.80	0.82	0.82	0.84
Average probability of assignment (range)	–	0.92–0.96	0.89–0.92	0.87–0.90	0.87–0.93	0.84–0.93	0.85–0.99
Vuong-Lo–Mendell–Rubin Likelihood Ratio Test (*p*-value)	–	0.0002	0.18	0.21	0.37	0.22	0.18
Lo–Mendell–Rubin Adjusted Likelihood Ratio Test (*p*-value)	–	0.0002	0.19	0.21	0.37	0.22	0.18
Group size (%)							
Group 1	100	69.45	46.64	37.67	7.60	14.47	15.18
Group 2		30.55	31.13	28.64	14.77	10.00	1.18
Group 3			22.23	13.60	33.16	27.20	6.50
Group 4				20.09	25.56	18.92	29.64
Group 5					18.91	6.57	21.10
Group 6						22.84	9.67
Group 7							16.74

**Figure 1:**
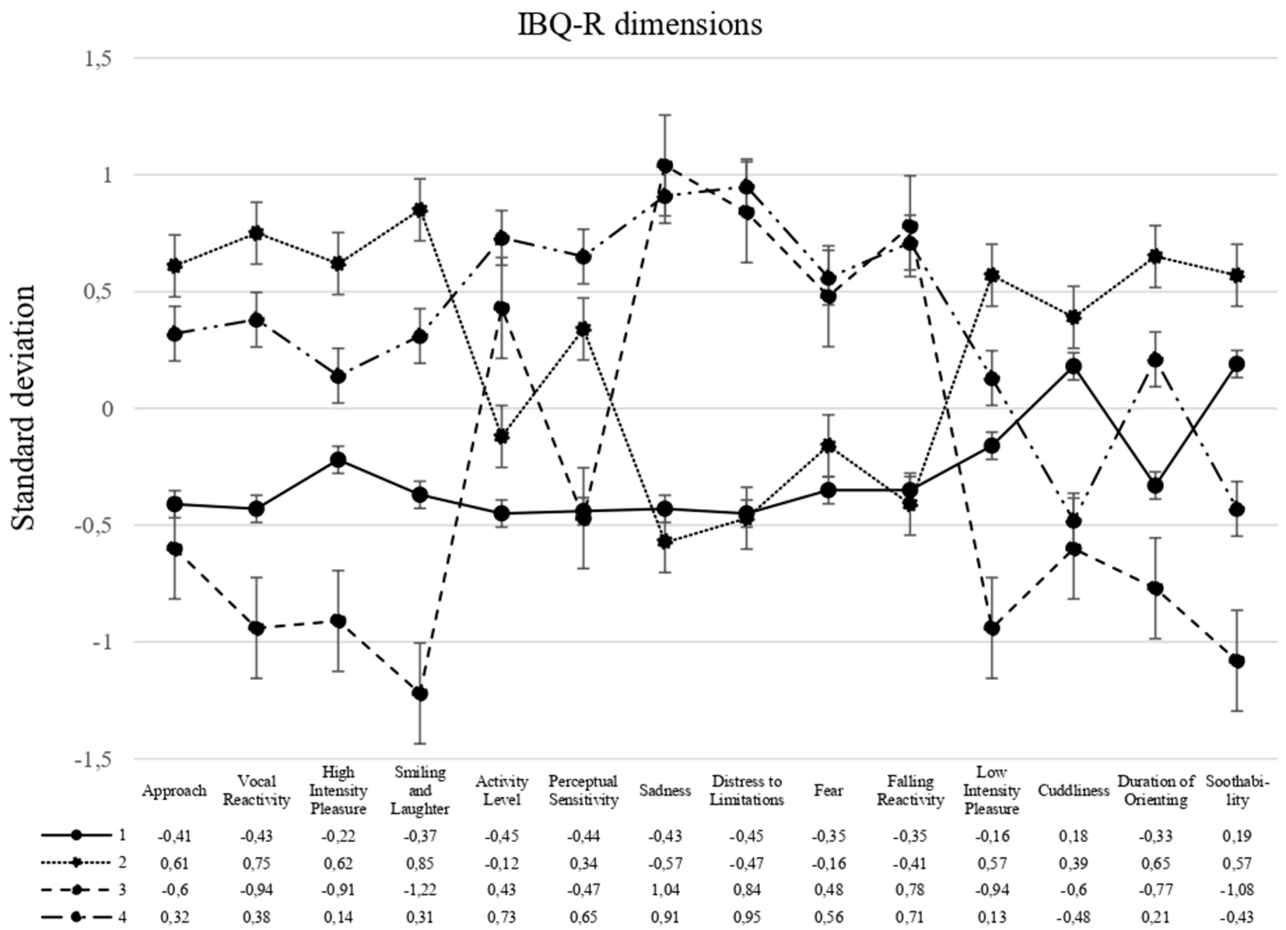
Temperament profiles based on the 14 dimensions of the IBQ-R (short form) for 6-month-old children (*N* = 433). Profile 1: Moderate Reactivity and Regulation (37.67%); Profile 2: High Positivity-High Regulation (28.64%); Profile 3: High Negativity-Low Regulation (13.60%); Profile 4: High Positivity and Negativity (20.09%).

*Z*-scores of each temperament dimensions for the four profiles are presented in Figure 1. Profile 1, referred to as *Moderate Reactivity and Regulation* (*n* = 163, 37.67%), was the most common profile in the sample. Infants in this group displayed slightly below-average values across the 14 dimensions studied, revealing minimal variation among the three factors, with slightly above-average values in the Orienting/Regulation factor for the dimensions of Cuddliness and Soothability. Profile 2, labeled as *High Positivity-High Regulation* (*n* = 124, 28.64%), had above-average values in the dimensions of the Surgency/Extraversion and of the Orienting/Regulation factors, and below-average scores in the dimensions of the Negative Affectivity factor, scoring low in Sadness and Distress to Limitations. Specifically, this profile differed from Profile 1, *Moderate Reactivity and Regulation*, by exhibiting higher values in Surgency/Extraversion and Orienting/Regulation. Profile 3, labeled as *High Negativity-Low Regulation* (*n* = 59, 13.60%), exhibited a predominance in the Negative Affectivity factor, with high values in the dimensions of Sadness, Distress to Limitations, Fear and Falling Reactivity. Additionally, this profile showed below-average scores in the Surgency/Extraversion factor (notably in Smiling and Laughter, High Intensity Pleasure, and Vocal Reactivity dimensions) and in the Orienting/Regulation factor (specifically in the Soothability and Low Intensity Pleasure dimensions). However, this profile exhibited above-average scores in the Activity Level dimension. Finally, Profile 4, labeled as *High Positivity and Negativity* (*n* = 87, 20.09%), displayed high values in all dimensions of both the Surgency/Extraversion factor (particularly in the dimensions of Activity Level and Perceptual Sensitivity) and the Negative Affectivity factor (notably in the dimensions of Sadness and Distress to Limitations). This profile showed moderate to low values in the Orienting/Regulation factor, with values slightly above average for the dimensions of Low Intensity Pleasure and Duration of Orienting, and below average values for Cuddliness and Soothability.

## Discussion

This study examined variations in infant temperament in a general population sample of 6-month-olds by employing LPA to identify distinct temperament profiles. The analysis revealed that a four-profile model provided the best fit for the data. The fine-grained examination of multiple temperament dimensions simultaneously is a significant contribution of this study since it allowed additional differentiation of infants that would not otherwise be distinguished based on a single temperamental factor. This study confirms that complex temperament profiles can be identified as early as 6 months of age, and also identifies a profile that has never been documented before. The four identified profiles were interpreted as follows: (1) *Moderate Reactivity and Regulation*, (2) *High Positivity-High Regulation*, (3) *High Negativity-Low Regulation*, and (4) *High Positivity and Negativity*.

The first profile, labeled as *Moderate Reactivity and Regulation*, emerged as the most prevalent in the sample with 37.67% of the infants being classified as such. Infants within this group demonstrated values slightly below-average across the dimensions examined, indicating minimal variation across the three primary temperament factors. This profile displayed slightly above-average scores in two dimensions of the Orienting/Regulation factor: Cuddliness - referring to the infant’s enjoyment of being held by a caregiver - and Soothability, which describes the reduction of agitation, crying, or distress when soothing techniques are provided by a caregiver. These values align with the relatively low scores observed for extraversion and negative emotionality, as regulatory skills may be solicited to a lesser extent. In a study by Dalimonte-Merckling and Brophy-Herb (2019), children identified to belong to an “easy” temperament - which had a description similar to the *Moderate Reactivity and Regulation* profile in the current study - exhibited lower levels of emotional reactivity, meaning they were less likely to display intense or negative reactions to environmental stimuli. Their behavior tended to be more consistent and predictable, reducing the demands placed on parents. As a result, parents of these children demonstrated higher levels of warmth and sensitivity in their interactions, effectively recognizing and meeting their children’s needs.

In another study by Prokasky et al. (2017), an “average cluster” profile was described as a group of children who exhibit moderate behaviors across all dimensions of temperament. This description resonates with the characteristics identified in the current profile *Moderate Reactivity and Regulation.* These children are not easily aroused and do not display marked distress or activity, but they are less likely to exhibit behaviors indicative of strong regulatory control. Prokasky et al. (2017) emphasized that children with such a moderate temperament can often go unnoticed or overlooked, both at home and in childcare settings. This inconspicuousness may lead to a lack of attention or encouragement, which could prevent these children from reaching their full potential. As such, it may be worthwhile for both childcare educators and parents to pay attention to this group to ensure that they remain engaged and motivated in educational and social settings and to consider proactive strategies to foster these children’s development.

The second identified profile, labeled as *High Positivity-High Regulation,* is characterized by particularly high scores in the Surgency/Extraversion factor, notably in Smiling and Laughter, reflecting frequent expressions of positivity. These infants also display elevated scores in the Orienting/Regulation factor, indicating a strong capacity to find enjoyment in calm activities and to recover easily from distress. In contrast, their scores in the Negative Affectivity factor are below average, with notably low levels in Sadness and Distress to Limitations. This relatively prevalent profile, representing nearly one-third of the infants in the sample (28.64%), represents characteristics mainly related to positive affectivity. In a sample of 54-month-old children, Laible et al. (2014) described a similar profile - with high regulation and low negative emotionality - which was associated with high levels of social competence, as well as prosocial and cooperative behaviors, as rated by teachers and parents. Children in this profile, due to their strong emotional regulation, would demonstrate an enhanced ability to manage their emotions, potentially allowing them to navigate social situations with greater ease. Their low level of negative emotionality could be beneficial in social interactions, facilitating cooperation and prosocial behaviors. Gartstein et al. (2017) also identified a profile with very similar characteristics, labeled as “High Positive/Regulated.” As in the current study, this profile was marked by high levels of temperament dimensions associated with Extraversion and Orienting/Regulation, combined with low levels of Negative Affectivity. According to the authors, this profile may confer a form of emotional resilience, allowing infants to engage in social situations in a more adaptive, approachable manner without the emotional burden typically associated with heightened negative reactivity. Such infants are generally perceived as pleasant and easygoing by those around them, thereby reinforcing positive interactions with parents and others. Given these temperamental dispositions, these infants can be viewed as highly engaged, positively responsive to parental cues, and displaying greater regulation and agreeableness, as well as lower negative reactivity, making them particularly receptive and accessible in social interactions. The identification of this profile within our sample of 6-month-old infants provides a valuable perspective on the early emergence of these positive emotional dispositions and their potential role in fostering social adaptability and relational harmony.

The third profile, identified as *High Negativity-Low Regulation*, is the least prevalent in this sample, representing 13.60% of the infants. These infants are characterized by heightened levels in dimensions associated with Negative Affectivity, particularly Sadness, Distress to Limitations, and Falling Reactivity, which reflect their sensitivity to emotional challenges and frustrations. In contrast, they exhibit below-average levels in traits related to the Surgency/Extraversion and Orienting/Regulation factors. However, their above-average Activity level stands out. Beekman et al. (2015) identified the “Negative Reactive” profile, characterized by high activity levels, heightened distress to novelty and limitations, and low levels of smiling, laughter, and soothing. Children in this profile tended to exhibit notable stability over time, particularly between the ages of 9 and 27 months, suggesting a likelihood of remaining within this temperament profile as they develop. This developmental consistency highlights the enduring combination of heightened negative emotionality and reduced self-regulation, persisting as children transition from infancy into early childhood. The work of Laible et al. (2014) can be a potential avenue for interpreting the *High Negativity-Low Regulation* profile identified in the present study. They describe a “Low regulation/High emotionality” profile, which is characterized by elevated levels of negative emotions (notably anger, sadness, and fear) and reduced capacities for voluntary control, including attention regulation and inhibitory control. In terms of social consequences, children with this profile were found to exhibit significantly lower prosocial and cooperative behaviors both at home and in school compared to children in other profiles. Furthermore, they demonstrated heightened vulnerability to strained social relationships, often marked by conflict or peer rejection. Given the shared dimensions between this profile and the current *High Negativity-Low Regulation* profile - particularly the combination of below-average regulation and high negative emotionality - these conclusions offer valuable insights into the potential social and developmental implications of the current findings. Preventative programs should pay attention to identifying and supporting these children.

The fourth profile, *High Positivity and Negativity*, describes infants with high levels in the dimensions of both the Surgency/Extraversion and the Negative Affectivity factors, representing 20.09% of the sample. These infants exhibit a particularly high activity level, including motor activity like arm and leg movements, and an increased perceptual sensitivity, indicating their ability to detect subtle environmental stimuli. Their positive reactivity is also evident through above average laughter and vocalization during daily activities and excitement in anticipation of pleasurable activities. Interestingly, this profile is further distinguished by high scores in Negative Affectivity, especially in the dimensions of Sadness, Distress to Limitations and Falling Reactivity. This means that, despite their activity and sociability, these infants tend to exhibit negative emotions when faced with personal discomfort or restrictions corresponding to a higher reactivity in both the Surgency/Extraversion and the Negative Affectivity factors. In addition, the dual presence of high sociability and negative emotional reactivity might seem contradictory, but it suggests a complex interplay between an active engagement with the environment and a heightened sensitivity to emotional distress. This combination of characteristics could reflect a heightened reactivity to both positive and negative stimuli, implying that these infants are highly responsive to their surroundings. For example, an infant can simultaneously be curious, active, and sociable, yet prone to negative emotional states when faced with challenging situations or limitations. These infants also display varied levels in the Orienting/Regulation factor, with slightly above average Low Intensity Pleasure (enjoyment of low intensity activities) and Duration of Orienting (sustained attention on a single object), and lower levels of Cuddliness (expressing pleasure and well-being when cuddled by their caregiver) and Soothability (difficulty calming down). These variations in the dimensions related to the Orienting/Regulation factor reflect both a positive tendency to be calm in certain situations, but difficulty being held and calmed down by caregivers.

To our knowledge, the *High Positivity and Negativity* profile has not been previously documented. There may be both developmental and methodological explanations for this. A possible developmental explanation could be related to the age of the children. While the majority of studies examining temperament profiles focus on toddlers to school-aged children, the present study focuses on young infants. The fact that the infants were only 6 months old may be associated with more intense emotional manifestations, in terms of both positive and negative reactivity, while their self-regulation skills are still emerging. Furthermore, the present study considered a significant number of distinct temperamental dimensions, which has not been done before. This could hence have contributed to the identification of this previously undocumented and more complex temperamental profile.

Previous research provides insights into the potential behavioral outcomes based on the temperamental characteristics of infants corresponding to the *High Positivity and Negativity* profile, suggesting that high negative emotionality can be problematic when not balanced with adequate self-regulation (Eisenberg and Fabes, 2006; Rydell et al., 2003). For example, children with high negative emotionality and low regulation are described as being the least likely to engage in prosocial and cooperative behaviors (Laible et al., 2014). However, this does not fully apply to our group, which also exhibits some above-average self-regulation aspects, specifically in the dimensions of Low Intensity Pleasure and Duration of Orienting. Accordingly, these infants will need to further develop their self-regulation strategies, but they show some helpful predisposition. While these predispositions could influence long-term developmental outcomes, the implications remain unclear, as this profile has not been previously described. Additionally, the combination of high extraversion and high negative emotionality presents an intriguing combination whose impact on development, whether it acts as a protective factor or a risk factor for these infants, remains to be determined. Given the limited literature on this type of profile, future studies are encouraged to examine the developmental trajectory of these children in greater detail to provide more nuanced insights into their progress and outcomes.

Infants within these four identified profiles demonstrate notable temperamental differences. Identifying these profiles at such an early age is particularly valuable, as it allows us to distinguish infants not solely on a single trait but through the combination of multiple dimensions. Furthermore, examining each temperament factor individually could have made it challenging to differentiate infants in the *High Negativity-Low Regulation profile* (Profile 3) compared to those in the *High Positivity and Negativity* (Profile 4), as both exhibit similar levels on all the dimensions of Negative Affectivity factor. However, their overall dispositions and needs differ significantly. Identifying these profiles also reflects the varied experiences parents may encounter during the early months of their infant’s life. The combination of temperamental traits in each profile may place varying demands on parents, requiring them to adapt their parenting strategies and mobilize specific resources to meet their child’s unique needs. This highlights the relevance of the concept of “goodness of fit,” as introduced by Chess and Thomas (1996; Thomas, 1977), which refers to the alignment between a child’s temperament and the characteristics or expectations of their environment. A “good fit” supports healthy development by fostering a harmonious interaction between the child and his environment, while a “poor fit” arises when the child’s behaviors are perceived as disruptive or difficult to manage by caregivers or others in their surroundings.

Applying this concept to the profiles identified in this study raise the possibility that specific parental behaviors can promote optimal child development. For instance, for infants in the *Moderate Reactivity and Regulation* profile, who exhibit low emotional and behavioral reactivity and moderate regulation, parents may naturally find it easier to respond with warmth and sensitivity, as these infants tend to be predictable and cooperative. In the *High Positivity-High Regulation* profile, infants display high emotional regulation and sociability, making them highly responsive to parental cues and adept at navigating social interactions. For example, parents can enhance their child’s development by encouraging their natural inclination for cooperation and creating opportunities for prosocial behaviors. Conversely, infants in the *High Negativity-Low Regulation* profile could present greater challenges, given their heightened negative emotions and lower regulation skills. These infants could benefit from parents providing emotional support, clear structure, and strategies to improve self-regulation and reduce distress. Finally, the *High Positivity and Negativity* profile, characterized by heightened emotional reactivity in both positive and negative dimensions, could require parents to find a balance between nurturing the infant’s enthusiasm and sociability while addressing their intense reactions to frustration or distress. Creating an adaptable environment that offers emotional regulation tools and opportunities for exploration may help these infants navigate their heightened reactivity and develop positive social behaviors. By aligning their approach to their child’s temperament, parents could help foster a better “goodness of fit” between the child and their environment, hence supporting their unique developmental needs and fostering positive outcomes.

### Strengths, limitations and future directions

This study has several strengths, particularly in its focus on a specific and underrepresented age group in temperament studies, namely infants 6 months of age. The identification of temperament profiles at such a young age represents a significant achievement, with multiple practical and scientific implications. Our study offers valuable new insights and serves as a useful starting point for further research in this area, such as identifying variables that predict these profiles and outcomes that can be linked to these profiles. The findings could also be used to inform parents on early child temperament. Being aware of these profiles and overall variations in child temperament can help normalize the experience parents may have regarding their child’s temperament, thereby potentially reducing parental stress and enhancing their sense of efficacy.

Despite these significant contributions, some limitations should be acknowledged. Firstly, although our sample size is considerable, expanding the sample size in future research could yield more definitive insights into the presence of temperament groups across a broader population. It is also important to note that the sample comes from relatively advantaged socioeconomic background and was ethnically homogeneous, which limits the generalizability of our findings. Further studies with more diverse samples could confirm if these four profiles apply across populations.

Additionally, due to our limited sample size, we did not examine profiles separately for boys and girls. Previous studies have established gender differences in temperament traits (de Boo and Kolk, 2007; Else-Quest et al., 2006; Gagne et al., 2013; Olino et al., 2013). Generally, boys tend to show higher levels of Surgency while girls present higher levels of Effortful Control (Else-Quest et al., 2006). Gender as well as other factors (e.g., sociodemographic and perinatal factors; Gouge et al., 2020; Werner et al., 2007) should be investigated to understand how they contribute to the membership in the different temperamental profiles.

Another limitation lies in the cross-sectional nature of our study, which limits the scope of our findings to a single point in time. Lastly, our results can only be generalized to children within the specific age range of our sample (3–12 months), and caution should be taken when extending these findings to other age groups. For example, self-regulation abilities are still developing at this age and could be measured more accurately as the child grows older. Moreover, both the stability and the predictive role of these profiles for later socio-emotional and cognitive outcomes throughout infancy and childhood should be examined. Targeted prevention and intervention programs could therefore be developed to address the unique temperamental profiles of children identified as being at-risk, thereby supporting their development more effectively.

## Conclusion

This study highlights the diversity of infant temperament profiles and emphasizes their potential role in shaping early development. By identifying distinct profiles such as *Moderate Reactivity and Regulation*, *High Positivity-High Regulation*, *High Negativity-Low Regulation* and *High Positivity and Negativity,* this study provides a more precise understanding of how multiple dimensions of temperament combine during infancy. These findings could be valuable in guiding preventive interventions with parents of young children, helping to normalize the diversity of temperament experiences and reducing potential parental stress related to perceived challenges in early childhood.

Future longitudinal research is needed to explore how these profiles evolve over time and how they influence later development. Additionally, identifying prenatal and early factors associated with these profiles could help uncover critical risk and resilience factors, further informing targeted interventions. Therefore, expanding this work to include more diverse and representative populations will also enhance its applicability and ensure a broader impact on understanding and supporting early childhood development.

## Data Availability

The datasets presented in this article are not readily available because the data that has been used is confidential. When agreeing to participate, participants were assured that raw data would remain confidential. Requests to access the datasets should be directed to Jessica Pearson, jessica.pearson2@uqtr.ca.
